# Nanoparticle-mediated dual delivery of resveratrol and DAP5 ameliorates kidney ischemia/reperfusion injury by inhibiting cell apoptosis and inflammation

**DOI:** 10.18632/oncotarget.17135

**Published:** 2017-04-17

**Authors:** Yong Xu, Bo Zhang, Da Xie, Yu Hu, Hai-Lun Li, Li-Li Zhong, Hong-Wu Wang, Wei Jiang, Zun-Ping Ke, Dong-Hui Zheng

**Affiliations:** ^1^ Department of Nephrology, The Affiliated Huai'an Hospital of Xuzhou Medical University and The Second People's Hospital of Huai'an, Huai'an, China; ^2^ Department of Dermatology, Hubei Provincial Hospital of TCM, Hongshan, Wuhan, China; ^3^ Department of Nephrology, The Secend Affiliated Hospital of Nanjing Medical University, Nanjing, China; ^4^ Department of Cardiology, The Fifth People's Hospital of Shanghai, Fudan University, Shanghai, China

**Keywords:** Res-DAP5 nanoparticles, apoptosis, inflammation, ischemia-reperfusion, acute renal injury

## Abstract

Ischemia reperfusion (I/R) injury is a leading cause of acute kidney injury with high morbidity and mortality due to limited therapy. NMDA receptor inhibitor (DAP5) and resveratrol (Res) could ameliorate kidney I/R injury, but their use is limited by low aqueous solubility and poor stability. Here, we examined the potential protective effects of Res-DAP5 nanoparticles (NP) against renal I/R injury. Mice were subjected to renal ischemia for 30 min followed by reperfusion for 24 h. The results showed that Res-DAP5-NP could decreased serum creatinine (Cr) and urea nitrogen (BUN), alleviated tubular damage and oxidative stress. In addition, Res-DAP5-NP suppressed cell apoptosis, promoted the expression of p-DAPK, and inhibited the expression of p-CaMK and p-AKT. Furthermore, Res-DAP5-NP decreased the production of pro-inflammatory cytokines such as tumor necrosis factor-α, IL-1β, IL-6, and p-IκBα induced by renal I/R injury. In addition, Res-DAP5-NP also attenuated renal I/R injury *in vivo*, as manifested by increase in cell viability, SOD level, and the expression of p-DAPK, decreases in intracellular Ca^2+^ concentration and the expression of p-CaMK. Taken together, our findings indicates that Res-DAP5-NP could effectively protect renal I/R injury by inhibiting apoptosis and inflammation responses, possibly through AKT/NMDA/CaMK/DAPK and NF-κB pathways.

## INTRODUCTION

Acute kidney injury incuced by ischemia/reperfusion (I/R) is a pivotal cause of acute renal failure with high morbidity and mortality [1]. However, there is limited therapy in clinical applications presently. Several pathogenesis cause to renal I/R injury including excitatory toxicity, intracellular Ca2+overload, oxidative stress, etc. Nevertheless, the precise mechanisms are not well understood. It has been reported that cell apoptosis and inflammation serves a key function in acute kidney injury incuced by I/R [2, 3]. Therefore, inhibiting apoptosis and inflammation may be an effective strategy to attenuate renal I/R injury.

N-methyl-d-aspartate (NMDA) is excitatory neurotransmitter involved in learning and memory formation. Recently, the role of NMDA is also discovered in peripheral organs such as bones, cardiovascular, and kidneys [4]. It has been reported that hyperactivity of NMDA receptors in kidneys leads to renal damage in rodents [5]. Antagonism of allosteric sites of NMDA receptor demonstrated renoprotection against I/R-induced acute kidney injury in rats [6]. Activation of NMDA receptor could cause calcium flux, activation of calcineurin, death-associated protein kinases (DAPK) dephosphorylation, and subsequent promote DAPK mediated death pathway, which is one of the main mechanisms of NMDA toxicity [7]. Kishino M et al have found that I/R stimuli enhanced DAPK catalytic activity, whereas DAPK deletion mutant attenuated kidney malfunction after I/R injury [8], which furtherly demonstrated the function of DAPK pathway in acute kidney injury incuced by I/R.

Oxidative stress has widely been recognized as one of the main pathogenesis in acute kidney injury incuced by I/R. Previous study showed that ROS could sensitize Ca^2+^/calmodulin-dependent kinase (CaMK) to Ca^2+^, which is upstream signal of DAPK [9]. More importantly, it have been reported that in response to oxidative stimuli, CaMKII activation might only need extremely low Ca2+/CaM levels [7]. So, we hypothesized that excessive ROS generated in the I/R-injured tissues might comprise the effect of NMDAR inhibitor, which is to say, cause NMDAR inhibitor “desensitization”. Recent studies showed that combined therapy using agents with different molecular mechanisms achieved better therapeutic effects than single drug strategies and at least partially counteracted drug resistance [10, 11]. Based on the above analysis, we proposed that NMDAR inhibitor combined antioxidants might serve as a new strategy for treatment of renal I/R injury.

Resveratrol (Res), a naturally occurring plant-derived molecule, was shown to have potent antioxidant properties. It has been reported that Res as a scavenger of free radical might improve acute kidney injury induced by I/R in rats [12]. However, *in vivo* studies on this regard have been limited by fast clearance and low bioavailability of Res [13]. Many studies have shown that nanoparticles can overcome these shortcomings to increase Res delivery [13–15]. Particularly, poly(ϵ-caprolactone)-poly(ethylene glycol) (PCL-PEG) copolymer has been widely served for the delivery of anticancer agents because of its perfect properties [16, 17]. Nevertheless, it is not known if PCL-PEG copolymers could serve for the delivery of kidney protectants. It is widely accepted that combined therapies against I/R injury might be more effective than monotherapy, which because it could function through several different mechanisms [18]. So, our research attempted to investigate the role of Res and NMDAR inhibitor (DAP5) co-delivered with nanoparticles on kidney I/R injury and the underlying mechanisms.

## RESULTS

### Characterization of Res-DAP5-NP

**Figure 1:**
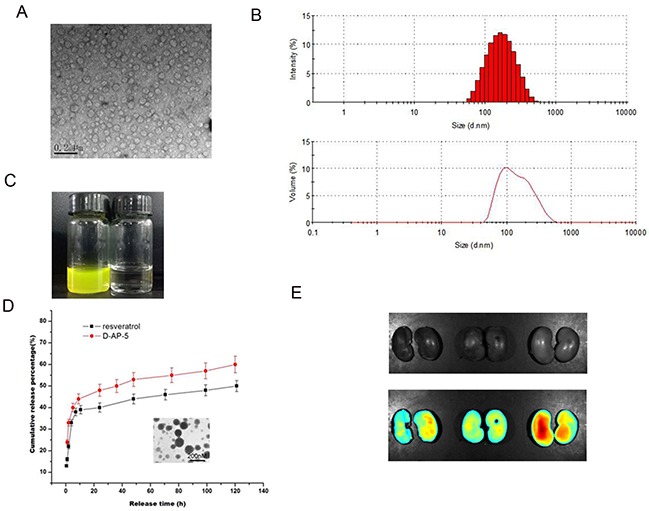
Characterization of Res-DAP5-NP **(A)** Morphology of Res-DAP5-NP. **(B)** Size and Zeta potential of Res-DAP5-NP. **(C)** Pictures of deionized water and solutions of Res-DAP5-NP. **(D)** Cumulative *in vitro* release profile of Res and DAP5 from Res-DAP5-NP. **(E)** Res-DAP5-NP gathered in the renal ischemia-reperfusion area. Data are expressed as mean ± SD (n= 3).

As shown in Figure 1, the morphology of Res-DAP5-NP was observed by transmission electron microscopy, with almost spherical shape at a size of around 200 nm. The water solution of Res-DAP5-NP shows a yellow color and is basically transparent. The mean size of Res-DAP5-NP was about 152 nm, while the surface charge of Res-DAP5-NP was slightly negative. Figure 1D shows a quick release of Res and DAP5, about 40%, within the first 5 hours, which may have resulted from the inevitable affiliation of certain drugs to the surface of Res-DAP5-NP. In the following process, Res and DAP5 was released in a controlled manner from the nanoparticles. It is clear that Res-DAP5-NP characterized by a sustained releasing pattern, could be a promising novel formulation of Res-DAP5-NP for future applications. We also found that there were obvious yellow-green fluorescence gathered in the renal I/R area.

### Effects of Res-DAP5-NP on renal function

**Figure 2:**
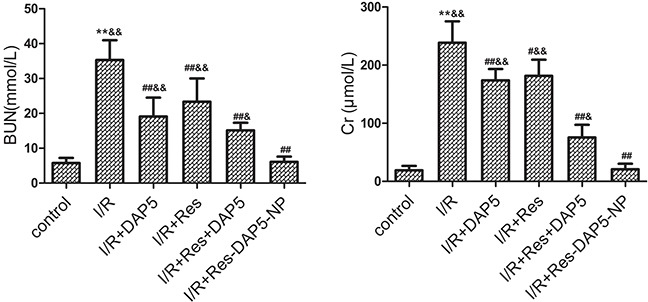
Effects of Res-DAP5-NP on serum levels of urea nitrogen (BUN) and Creatinine (Cr) in rats induced by renal I/R injury Data are expressed as mean ± SD (n= 3). ^*^P< 0.01 vs the control group; ^#^*P*<0.05, ^##^*P*<0.01 vs the I/R group; ^&^*P*<0.05, ^&&^*P*<0.01 vs the Res-DAP5-NP group.

As shown in Figure 2, kidney I/R injury cause a sharp raise in the contents of BUN and Cr. However, the levels of BUN and Cr in the Res, DAP5, Res+DAP5, and Res-DAP5-NP groups decreased significantly. In addition, the levels of BUN and Cr in Res-DAP5-NP group were obviously lower than those in the Res, DAP5, and Res+DAP5 groups. These data indicated that Res-DAP5-NP could attenuate renal dysfunction following renal I/R injury.

### Effect of Res-DAP5-NP on renal histopathological changes

**Figure 3:**
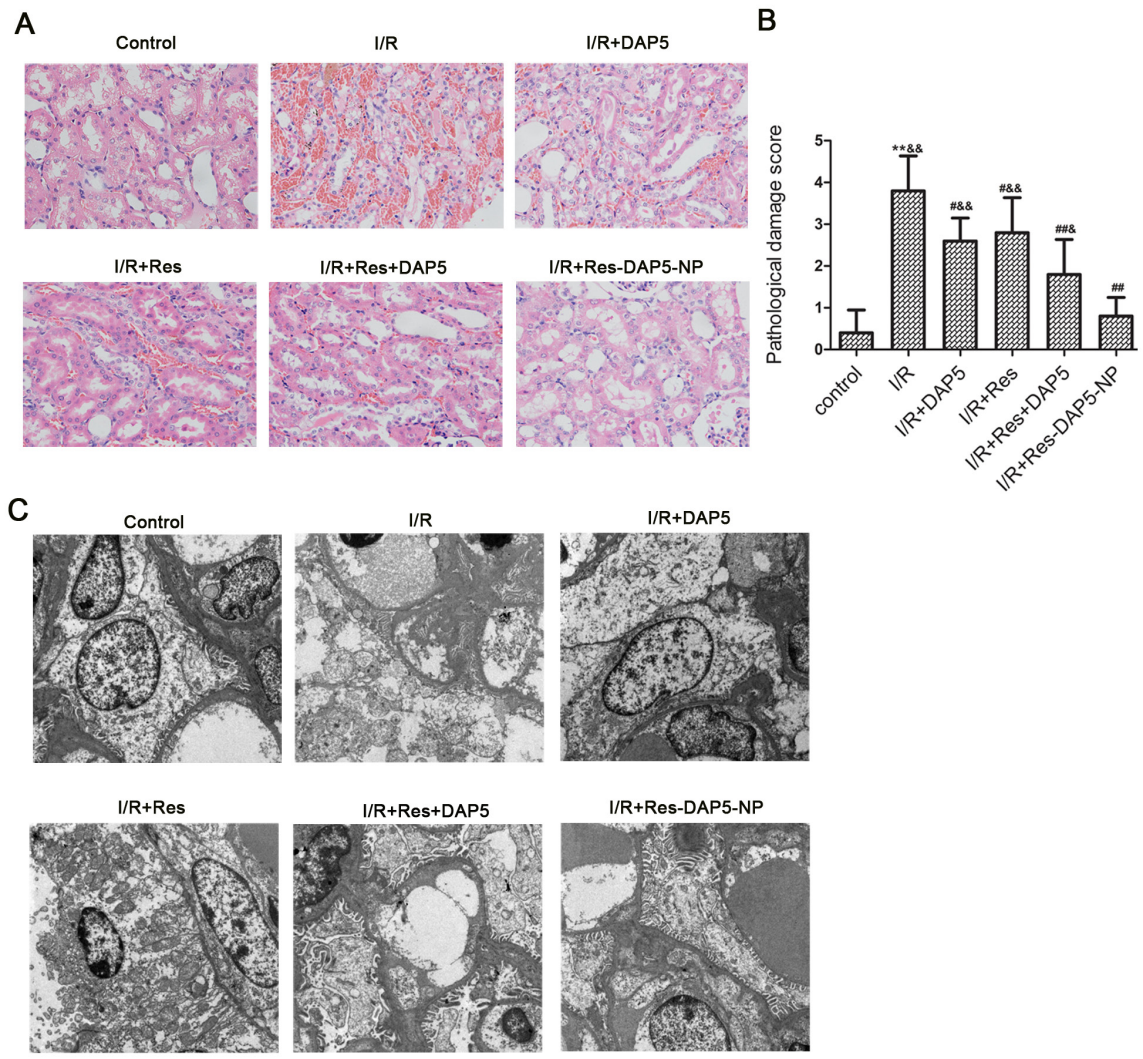
Histopathologic evaluation of kidney I/R injury **(A)** Light microscope images (×400). Semi-quantitative injury scores, expressed as the mean±SD (n= 5), are shown in **(B)** ^*^P < 0.01 vs the control group; ^#^*P*<0.05, ^##^*P*<0.01 vs the I/R group; ^&^*P*<0.05, ^&&^*P*<0.01 vs the Res-DAP5-NP group. **(C)** Transmission electron microscopy analysis of the effect of Res-DAP5-NP on renal I/R injury. Micrographs shown were taken at ×10,000 magnification.

As shown in Figure 3A, histological alterations such as tubular dilatation, cytoplasmic spaces, and cell necrosis were observed in the I/R group. However, compared to the I/R group, there were less alterations in the Res, DAP5, Res+DAP5, and Res-DAP5-NP groups, with the least being in Res-DAP5-NP group. Similarly, as shown in Figure 3A by transmission electron microscopy, cells lost their normal morphology, and microvilli shed into the lumen following I/R injury. However, the Res, DAP5, Res+DAP5, and Res-DAP5-NP groups exhibited less damage to cellular morphology, with Res-DAP5-NP group being the least damaged. These results demonstrated that Res-DAP5-NP pretreatment could decrease renal pathological damage induced by I/R injury.

### Effects of Res-DAP5-NP on oxidative stress

**Figure 4:**
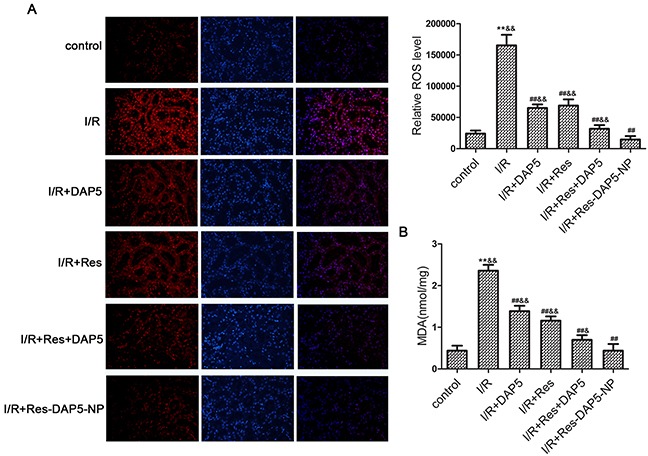
Effects of Res-DAP5-NP on oxidative stress in rats induced by renal I/R injury **(A)** The ROS generation. Data are expressed as mean ± SD (n= 3). **(B)** The level of MDA contents.^*^P < 0.01 vs the control group; ^#^*P*<0.05, ^##^*P*<0.01 vs the I/R group; ^&^*P*<0.05, ^&&^*P*<0.01 vs the Res-DAP5-NP group.

As shown in Figure 4, compared with the control group, the levels of ROS and MDA of renal tissues in the I/R group markedly increased. Pretreatment with Res, DAP5, Res+DAP5, and Res-DAP5-NP significantly reduced the contents of ROS and MDA in kidney tissues. Moreover, the contents of ROS and MDA in Res-DAP5-NP group were lower than those in the Res, DAP5, and Res+DAP5 groups. These data implied that Res-DAP5-NP pretreatment can suppress oxidative stress induced by renal I/R injury.

### Effects of Res-DAP5-NP on cell apoptosis

**Figure 5:**
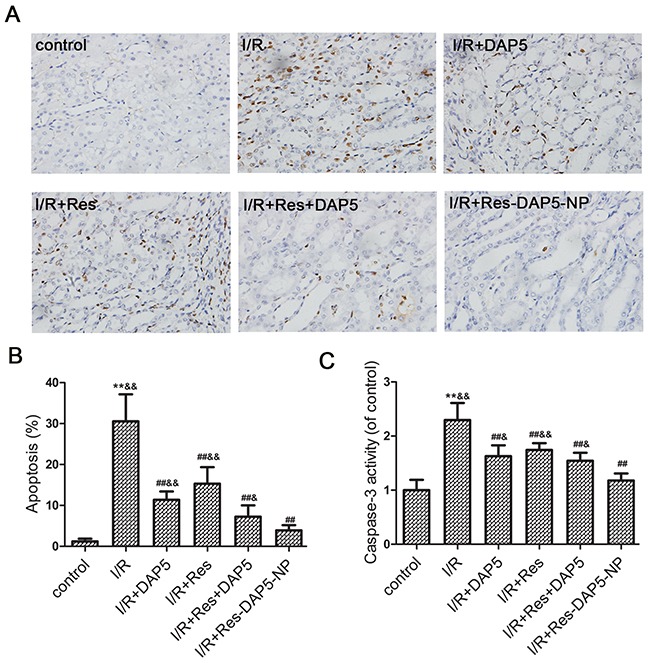
Effects of Res-DAP5-NP on cell apoptosis induced by renal I/R injury **(A)** Representative images (magnification, ×400) of renal TUNEL assay from rats subjected to renal I/R. **(B)** The percentage of TUNEL-positive cells in the kidney sections increased following I/R. **(C)** Effects of Res-DAP5-NP on caspase 3 activity in kidney tissues after I/R injury, quantitatively estimated as the fold-change relative to the I/R group. Data are expressed as mean ± SD (n= 3). ^*^P<0.01 vs the control group; ^#^*P*<0.05, ^##^*P*<0.01 vs the I/R group; ^&^*P*<0.05, ^&&^*P*<0.01 vs the Res-DAP5-NP group.

Apoptosis is another cause of renal injury following I/R. As shown in Figure 5, there were many apoptotic cells in the I/R group as indicated by TUNEL assay. On the contrary, Res, DAP5, Res+DAP5, and Res-DAP5-NP significantly reduced the amount of apoptotic cells. However, the apoptotic cells number in Res-DAP5-NP group was fewer than those in the Res, DAP5, and Res+DAP5 groups. Furtherly, we found that caspase-3 activity increase markedly following I/R injury. In contrast, the caspase-3 activity in the Res, DAP5, Res+DAP5, and Res-DAP5-NP groups following I/R was reduced, while the least in the Res-DAP5-NP group. These data implied that Res-DAP5-NP pretreatment suppressed apoptosis upon renal I/R injury.

### Effect of Res-DAP5-NP on expression of p-CaMK, p-DAPK, and p-AKT

**Figure 6:**
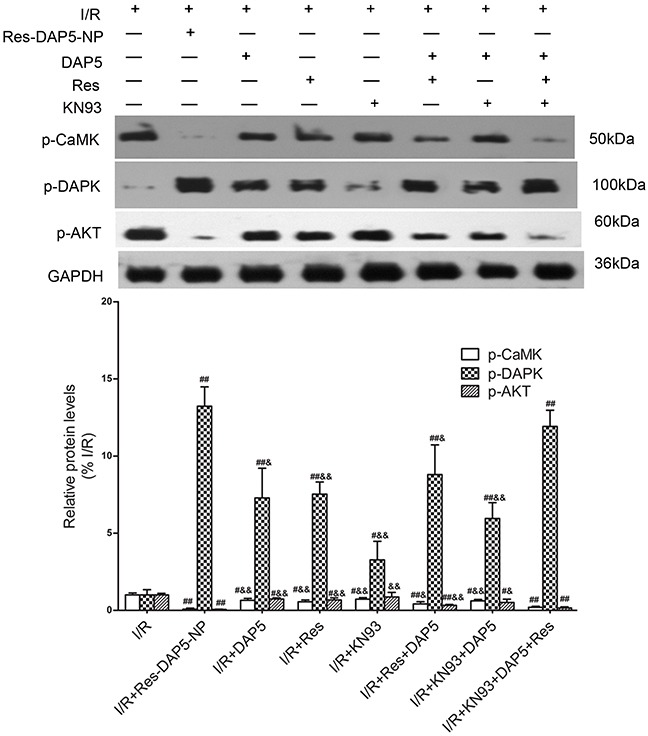
Effects of Res-DAP5-NP on expression of p-CaMK, p-DAPK, and p-AKT in rats induced by renal I/R injury Data are expressed as mean±SD (n= 3). ^#^*P*<0.05, ^##^*P*<0.01 vs the I/R group; ^&^*P*<0.05, ^&&^*P*<0.01 vs the Res-DAP5-NP group.

Western blot showed that comparing with the I/R group, the expression levels of p-DAPK in the Res, DAP5, Res+DAP5, and Res-DAP5-NP groups remarkably increased, and were most in Res-DAP5-NP group. Interestingly, comparing with the I/R group, the expression levels of p-CaMK and p-AKT were lower in the Res, DAP5, Res+DAP5, and Res-DAP5-NP groups obviously reduced, while were least in Res-DAP5-NP group (Figure 6).

### Effect of Res-DAP5-NP on inflammatory factor

**Figure 7:**
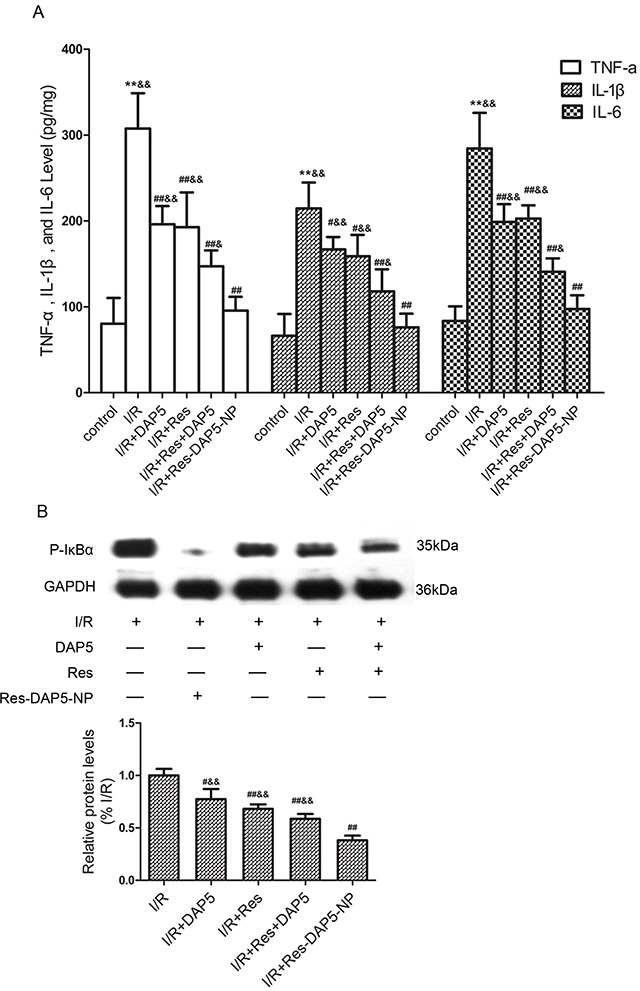
Effects of Res-DAP5-NP on pro-inflammatory factors **(A)** and phosphorylation of IκBα **(B)** in rats induced by renal I/R injury Data are expressed as mean±SD (n= 3). ^*^P<0.01 vs the control group; ^#^*P*<0.05, ^##^*P*<0.01 vs the I/R group; ^&^*P*<0.05, ^&&^*P*<0.01 vs the Res-DAP5-NP group.

As shown in Figure 7A, I/R injury can induce an obvious increase in TNF-α, IL-1β, and IL-6. Comparing with the I/R group, TNF-α, IL-1β, and IL-6 in Res, DAP5, Res+DAP5, and Res-DAP5-NP groups decreased significantly. Furthermore, the expression levels of these factors in Res-DAP5-NP group were obviously less than those in the Res, DAP5, and Res+DAP5 groups.

NF-κB is a well-known regulator of the transcription of inflammatory gene including TNF-α, IL-1β, and IL-6 during acute kidney injury. We also studyed the phosphorylation of IκBα. Upon exposure to Res-DAP5-NP, the p-IκBα was significantly reduced. These results revealed that Res-DAP5-NP suppressed inflammatory response induced by kidney I/R injury.

### Effect of Res-DAP5-NP on renal H/R injury *in vitro*

**Figure 8:**
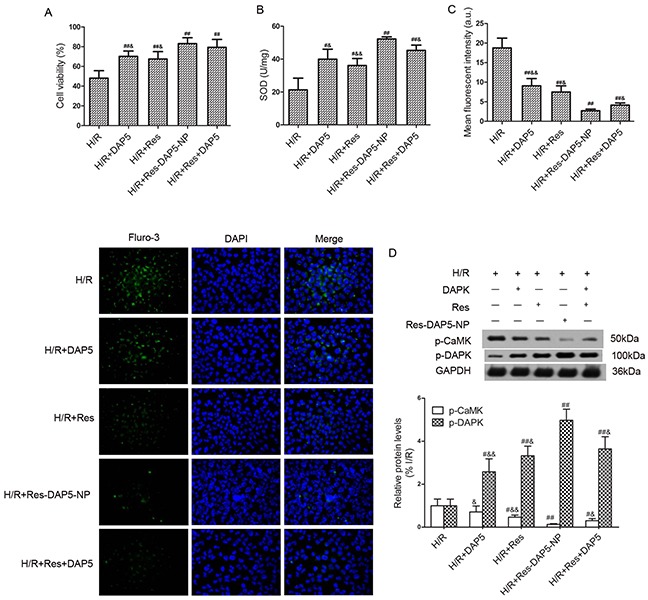
Effect of Res-DAP5-NP on renal I/R injury *in vitro* **(A)** cell viability was assessed by MTT assay. **(B)** Effects of Res-DAP5-NP on SOD levels after renal I/R injury. **(C)** Effect of Res-DAP5-NP on fluorescence intensity of intracellular Ca^2+^ after renal I/R injury. **(D)** Effects of Res-DAP5-NP on expression of p-CaMK and p-DAPK after renal I/R injury. Data are expressed as mean±SD (n= 3). ^#^*P*<0.05, ^##^*P*<0.01 vs the I/R group; ^&^*P*<0.05, ^&&^*P*<0.01 vs the Res-DAP5-NP group.

As shown in Figure 8A, H/R decreased cell viability, which was reversed to some degree by Res-DAP5-NP (10 μM). Furthermore, the activities of SOD significantly increased by Res-DAP5-NP compared with that in the H/R group (Figure 8B). Consistent with the results on enzymes, Res-DAP5-NP significantly reduced the intracellular Ca^2+^ concentration compared with that in the H/R group by fluo-3/AM (Figure 8C). To determine whether the CaMK-DAPK pathway was activated by Res-DAP5-NP, we measured the phosphorylated CaMK and DAPK, the results of which showed that the p-DAPK level increased remarkably and the p-CaMK level reduced significantly in the Res-DAP5-NP group (Figure 8D). Our findings as a whole lend to the conclusion that Res-DAP5-NP could prevent ischemia-reperfusion acute kidney injury *in vitro*.

## DISCUSSION

Apoptosis and inflammation were reported as crucial mechanisms underlying kidney I/R injury, and thus potential therapeutics against renal injury [2, 3].

Res and DAP5 are potent kidney protectants through many mechanisms to alleviate I/R injury. However, their efficacies are limited due to poor water solubility and challengings in target delivery. Previous studies have shown that nanoparticles can overcome these shortcomings [16]. In this study, we constructed co-delivered DAP5 and Res using PCL-PEG nanoparticles and aimed at investigating the effects of Res-DAP5-NP on renal I/R injury.

In our research, we firstly observed that Res-DAP5-NP significantly decreased kidney dysfunction and histopathologic changes following I/R, demonstrating the protection of Res-DAP5-NP in acute kidney injury.

To explore the underlying mechanisms, we examined the influence of Res-DAP5-NP on oxidative stress. In this study, we demonstrated that Res-DAP5-NP reversed the elevated MDA content and ROS accumulation *in vivo*, and recovered the levels of SOD *in vitro* upon renal I/R injury, suggesting that Res-DAP5-NP attenuate acute kidney injury induced by I/R possibly through its antioxidant properties.

Apoptosis is a vital pathological processes in acute kidney injury induced by I/R [2, 3], which was significantly inhibited by Res-DAP5-NP. In addition, Res-DAP5-NP promoted the expression of p-DAPK and inhibited the expression of p-CaMK and p-AKT *in vivo*. Furthermore, Res-DAP5-NP also attenuated renal I/R injury *in vitro*, manifested by increased cell viability, SOD level, and expression of p-DAPK, reduced intracellular Ca^2+^ concentration and expression of p-CaMK. Taken together, our findings indicates that Res-DAP5-NP could effectively protect acute kidney injury induced by I/R through AKT/NMDA/CaMK/DAPK signaling.

There is growing evidence that inflammation erves a key function in acute kidney injury induced by I/R [2, 3, 20]. Our research demonstrated that Res-DAP5-NP decreased the levels of TNF-α, IL-1β, and IL-6 in renal tissue and represents a feasible method to reduce inflammation-related injury. Furthermore, NF-κB is also a well-known regulator of the transcription of inflammatory gene during acute kidney injury [21]. Herein, we demonstrated that NF-κB involved in inflammation-related injury. These results suggest that Res-DAP5-NP could suppressed inflammatory response induced by kidney I/R injury via inhibiting activation of NF-κB.

In conclusion, our research showed that Res-DAP5-NP can protect rats against acute kidney injury induced by I/R through its anti-apoptosis and anti-inflammatory activities, possibly via AKT/NMDA/CaMK/DAPK and NF-κB pathway. Further studies in human clinical trials are necessary to exploit Res-DAP5-NP as a possible drug for the prevention of acute kidney injury.

## MATERIALS AND METHODS

### Construction of Res-DAP5 nanoparticles (Res-DAP5-NP)

Res-DAP5-NP were prepared as follows. Briefly, an amount of Res, DAP5 and PVP-b-PCL diblock copolymer were dissolved in acetone. The obtained organic solution was added dropwise into distilled water (ten times the volume) under moderate stirring at 25°C. Subsequently, the solution was dialyzed in a dialysis bag to thoroughly remove the acetone. Finally, solutions of drug-loaded nanoparticles was lyophilized for further utilization. Green fluorescent coumarin-6 was incorporated into the NPs to visualize the uptake by cells.

### Animals

Male Sprague-Dawley rats, aged 8-10 weeks (290-300g), were obtained from HFK Bio-Technology Co. Ltd. (Beijing, China). Animals were housed in a 12 h dark/light cycle animals facility with controlled temperature and humidity for at least 1 week. All procedures involving animal use were in accordance with the guidelines for care and use of experimental animals and were approved by the institutional ethics committee at Huai'an Second Hospital Affiliated to Xuzhou Medical College.

### *In vivo* renal I/R injury model

The rat model of renal I/R injury was induced by procedures similar to those described previously [19]. Briefly, rats were anesthetized with intraperitoneal injections of pentobarbital (50 mg/kg) and a midline abdominal incision was made. Renal ischemia was performed by clamping both renal vessels for 30 min. Then, the clamps were removed to reinitiate renal blood flow. Sham-operated rats underwent the same surgical procedure without clamping. After 24hrs recovery, rats were sacrificed, blood and tissue were harvested for analysis. To determine the effect of drugs, rats were treated with Res (2 mg/kg), DAP5(a NMDAR inhibitor, 2 mg/kg), KN93(a CaMKII inhibitor, 1 mg/kg) and Res-DAP5 nanoparticles (2 mg/kg) by tail vein injection 3 h before I/R surgery.

### Assessment of renal function

After reperfusion for 24 h, blood samples were collected. Serum creatinine (Cr) and blood urea nitrogen (BUN) levels were measured using commercially available kits (Jiancheng Pharmaceuticals, Nanjing, China) according to the manufacturer's instructions.

### Histological analysis

One-half of the removed renal tissue were fixed in 4% paraformaldehyde, embedded with paraffin, and sectioned into slides (4 μm). Then, the renal sections were subjected to hematoxylin-eosin (HE) staining for histological evaluation. The histopathological changes were evaluated by a pathologist in a blinded fashion according to the degree of tubular necrosis, hemorrhage and cast formation, as follows: 0, no change; 1, change affecting less than 25 % of the field; 2, change affecting 25–50 % of the field; 3, change affecting 50–75 % of the field; 4, change affecting more than 75 % of the field [19]. The other half of kidney samples were also observed by transmission electron microscope (Hitachi, Tokyo, Japan) under uranyl acetate and lead citrate double staining.

### Assays for ROS accumulation

The fluorescent probe 2′ 7′–dichlorodihydrofluorescein diacetate (DCFH-DA) was used to monitor the intracellular accumulation of ROS. Fresh frozen sections were incubated with 10 μM DCFH-DA for 25 min at 37°C in the dark. After rinsed two times with PBS, the sections were subjected to fluorescence analysis under a fluorescence microscope (Olympus, Japan). The mean densities of fluorescent images were quantified with Image Pro Plus software (Media Cybernetics).

### Detection of SOD and MDA content

In order to estimate the anti-oxidative activities of Res-DAP5-NP, the levels of SOD and MDA were determined by commercial available kits from Nanjing Jiancheng Bioengineering according to manufacturer's protocols.

### TUNEL assay

Renal samples were fixed in 4% paraformaldehyde, embedded with paraffin, and sectioned into slides (4 μm). Apoptosis was detected by using the TUNEL kit (Roche Diagnostics, Annheim, Germany) in accordance with the kit protocols. TUNEL slides were photographed with a microscope and the percentage of TUNEL-positive cells were counted at a magnification of ×400 for 5 fields per section.

### Caspase-3 activity assay

Renal tissue was homogenized in lysis buffer. Then, reaction buffer and caspase-3 substrate peptide were mixed in 96-well plates at 37°C for 4 h in the dark. After incubation, a microplate reader (excitation/emission 360/460 nm) was used to analyzethe samples.

### ELISA assay

The protein levels of tumor necrosis factor-α (TNF-α), interleukin (IL)-1β, and IL-6 in plasma were measured by ELISA according to the manufacturer's instructions (Biosource International Inc, United States).

### Cell culture and hypoxia/reoxygenation (H/R) model

Human proximal tubular cells (HK-2) were purchased by the Cell Bank of the Chinese Academy of Sciences (Shanghai, China). Cells were grown in DMEM supplemented with 10% fetal bovine serum at 37°C in a 5% CO2 incubator. For hypoxic treatment, HK-2 cells were incubated for 8 h in a atmosphere containing 1% O2, 5% CO2 and 94% N2, followed by reoxygenation (95% O2, 5% CO2,) for 24 h.

### Cell viability assay

MTT assy was used to measure cell viability. Briefly, HK-2 cells were seeded in 96-well plates at 5×10^3^ cells per well and incubated overnight to achieve 70% confluency. Next, cells were incubated with indicated drugs for 2 h prior to exposure to H/R injury. After drug treatments, 5mg/ml MTT solution was added, and incubated for 4h. Then, the medium was removed, and the dark blue crystals were dissolved in 150 μl DMSO. The absorbance at 570nm was measured.

### Measurement of intracellular calcium fluorescence intensity

HK-2 cells were incubated with 4 μM Fluo-3/AM in the dark for 40 min at 37°C. After dyeing, the cells were washed three times with PBS. Then, the images of Ca^2+^ concentration were monitored by the confocal microscope (Olympus, LSM, Japan) and fluorescence intensity was detected using FV10-ASW software.

### Western blot

Western blot analysis was performed as described previously [19]. In brief, protein samples were electrophoretically transferred onto a polyvinylidene fluoride membranes. Blots were blocked with 5% fat-free milk for 2 h at room temperature, incubated with primary antibodies against p-AKT (CST; 1:500), p-DAPK (Abcam; 1:500), p-CaMK (CST; 1:500), and IκBα (CST; 1:500) at 4°C overnight, and followed by incubation with HRP-linked secondary antibody for 1.5h. The results were analyzed by gray level of chemiluminescence.

### Statistics analysis

Differences among groups were statistically tested by one-way analysis of variance (ANOVA) using SPSS 16.0. Statistical significance was defined as *P*<0.05. All experiments were repeated three times, and all data were expressed as mean ± standard deviation (SD).
